# Community‐acquired *Stenotrophomonas maltophilia* bacteremia in liver cirrhosis: A case report

**DOI:** 10.1002/ccr3.7920

**Published:** 2023-09-19

**Authors:** Kentaro Shirakura, Masaji Saijo, Tatsuya Yamada, Myeongcheol Song, Shadia Constantine

**Affiliations:** ^1^ Sapporo Tokushukai Hospital Sapporo Japan

**Keywords:** bacteremia, community‐acquired, liver cirrhosis, *Stenotrophomonas maltophilia*

## Abstract

*Stenotrophomonas maltophilia* is a Gram‐negative bacterium, usually considered a nosocomial pathogen. Its role in community‐acquired infections has been reported, but it is still not typically included in differential diagnoses of patients not exposed to the healthcare system. Recently, some reports suggested that liver diseases might also act as a possible risk factor for community‐acquired *S. maltophilia* bloodstream infection. We report a case of a 77‐year‐old woman with a history of cirrhosis who was diagnosed with community‐acquired *S. maltophilia* bloodstream infection. *S. maltophilia* not only causes hospital‐acquired infections but is also emerging as a pathogen in community settings. Although community‐onset infection is still rare and might have lower mortality, this antibiotic‐resistant bacterial species should be considered a possible pathogen in patients with liver cirrhosis. Although trimethoprim‐sulfamethoxazole is considered the first‐line treatment, a study in vitro and a 4‐year review of *S. maltophilia* susceptibility in our institution found that the bacteria were more susceptible to minocycline than to trimethoprim‐sulfamethoxazole. Therefore, minocycline might become the first‐line treatment in the future.

## 1 BACKGROUND

*Stenotrophomonas maltophilia* (*S. maltophilia*) is a Gram‐negative bacterium that has recently gained importance due to its increasing frequency in nosocomial infections.^1^ Because of its low virulence and multidrug‐resistance, *S. maltophilia* is considered an opportunistic and nosocomial pathogen.^2^ Its role in community‐acquired infections has been reported, but it is still not typically included in differential diagnoses of patients not exposed to the healthcare system. Risk factors for *S. maltophilia* infection include underlying malignancy, presence of indwelling devices, immunocompromised status, cystic fibrosis, prior use of antibiotics, and long‐term stay in the hospital or intensive care unit (ICU).^2^ Patients with liver cirrhosis are susceptible to many bacterial infections, mostly Enterobacteriaceae such as *Escherichia coli* and *Klebsiella*. Recently, liver cirrhosis and liver malignancy patients have been identified as possibly at risk for *S. maltophilia* infections.^2^ *S. maltophilia* is intrinsically resistant to beta‐lactams, including penicillins, cephalosporins, aztreonam, and carbapenems, which are the usual empiric antibiotics used to treat infections in liver disease patients.^1^ We report a case of a 77‐year‐old woman with a history of alcoholic liver cirrhosis who was diagnosed with community‐acquired *S. maltophilia* bloodstream infection (BSI).

## 2 CASE REPORT

**FIGURE 1:**
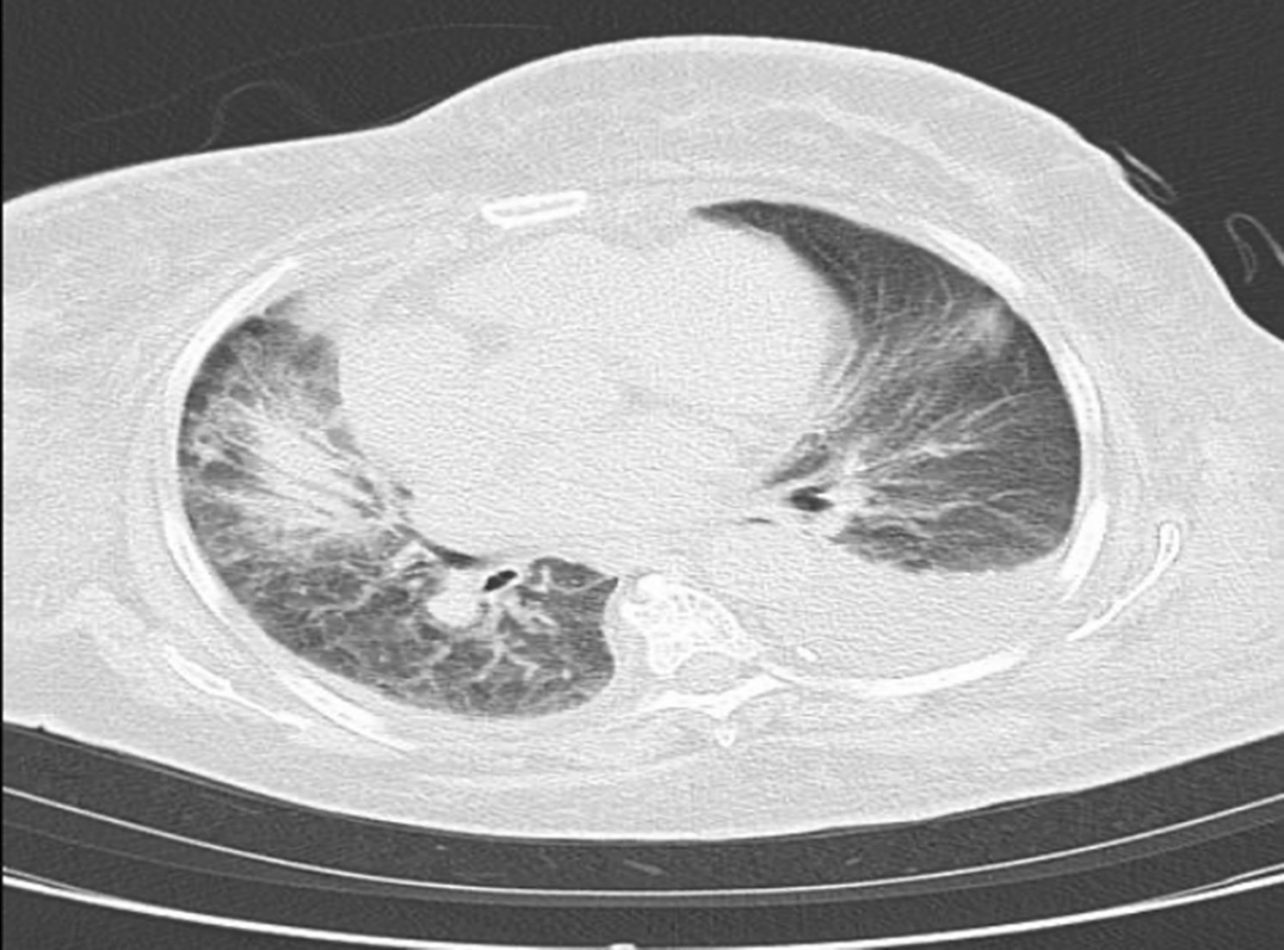
Chest CT finding.

**FIGURE 2:**
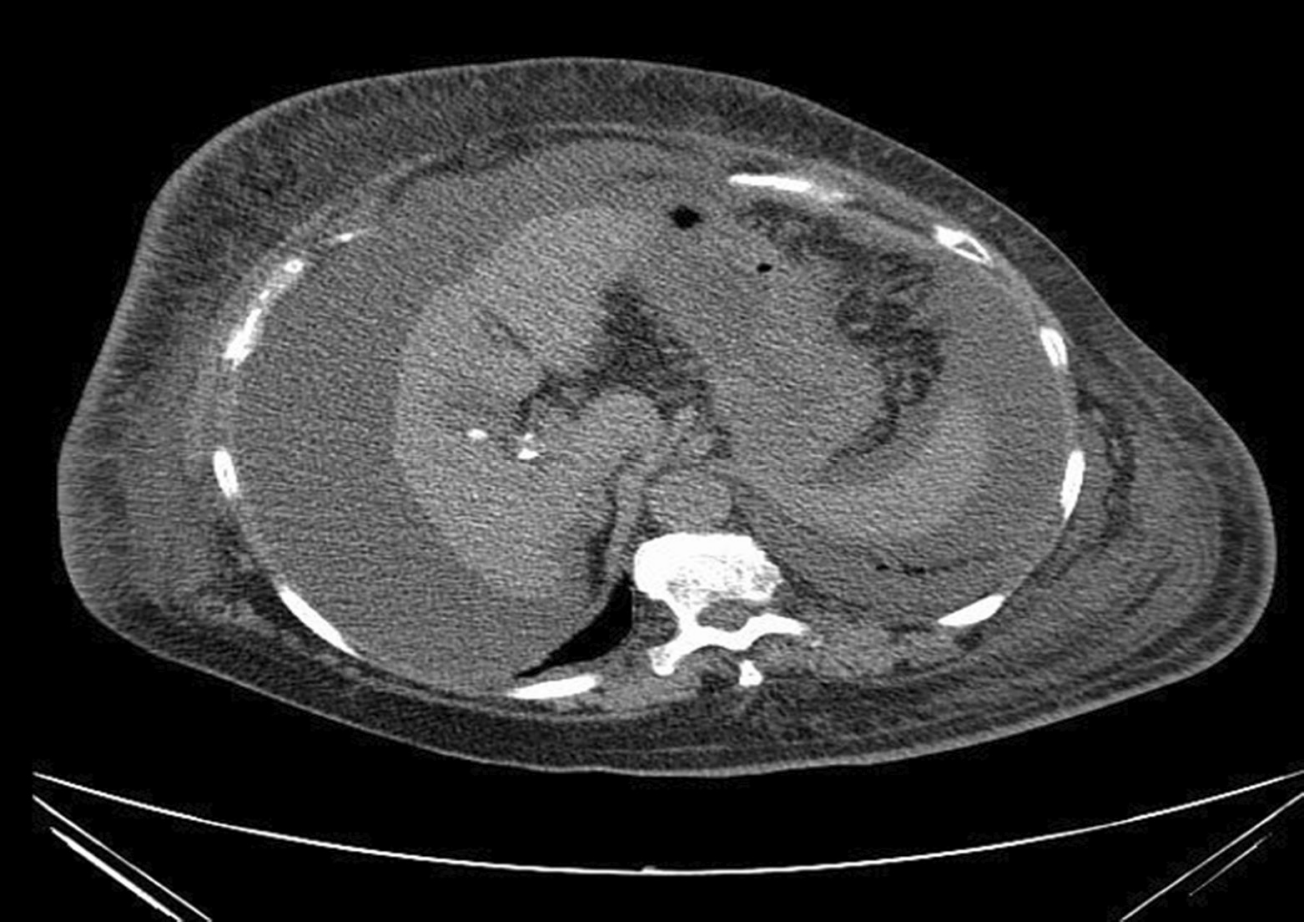
Abdominal CT finding.

A 77‐year‐old woman with a history of alcoholic liver cirrhosis presented to our institution complaining of progressive edema, fatigue, and generalized weakness over 3 months. She denied having regular health exams, prior hospitalizations, recent use of antibiotics, and previous diagnoses of diabetes mellitus or chronic respiratory disease. She also denied contact with animals or river water. The physical exam revealed a heart rate of 80 beats/min, blood pressure of 103/30 mmHg, oxygen saturation of 96% (room air), respiratory rate of 18 breaths/min, temperature of 33.4°C and a Glasgow Coma Scale (GCS) of 12 (E4V3M5). She had icteric sclera, abdominal distension, peripheral edema, and petechiae. A computed tomography (CT) showed ground glass opacities and pleural effusions bilaterally (Figure 1), atrophy of the left lobe of the liver, and ascites (Figure 2). White blood cell count was 5300/μL, and C‐reactive protein was elevated at 4.29 mg/dL. AST and ALT were 152 and 88 U/L, respectively. Total bilirubin 7.6 mg/dL, Alubumin 2.3 g/dL and PT‐INR 2.90. Creatinine 1.6 mg/dL and BUN 39.9 mg/dL. Her Child‐Pugh Score was class C.

She was initially diagnosed with possible bacterial pneumonia and received empirical treatment with ampicillin/sulbactam (ABPC/SBT). By hospital Day 4, her kidney function had declined significantly, requiring hemodialysis. On hospital Day 7, blood cultures identified *S. maltophilia* BSI, and the antibiotic regiment was changed to levofloxacin (LVFX) 250 mg intravenously every 8 h. Unfortunately, her multiorgan failure progressed, and despite aggressive supportive care, she died.

## 3 DISCUSSION

*Stenotrophomonas maltophilia* is a multidrug‐resistant, aerobic, Gram‐negative bacillus, previously known as *Pseudomonas* and *Xanthomonas maltophilia*. It is a ubiquitous inhabitant of soil, water, animals, and even food.^2^

Although *S. maltophilia* is considered to have limited pathogenicity, it is increasingly recognized as responsible for severe nosocomial infections, such as pneumonia, bacteremia, urinary tract infection, ocular infection, skin and soft tissue infection, endocarditis, cholecystitis, and meningitis in immunocompromised hosts.^1^, ^3^ In addition, infections can be further complicated by biofilm formation.^1^ The increased occurrence of *S. maltophilia* nosocomial infections is thought to be primarily due to the inappropriate use of antibiotics.

*S. maltophilia*'s importance in community‐acquired infection is seldom highlighted. Moreover, community‐acquired *S. maltophilia* bloodstream infections are relatively rare; therefore, empiric antibiotic regimens do not usually cover this bacterium. Previous studies describing community‐acquired *S. maltophilia* infections concluded that patients usually had comorbidities, especially malignancy.^2^ Chang et al. reported that in patients with *S. maltophilia* bacteremia, those with liver cirrhosis, liver metastases, and high Pitt bacteremia score (PBS) had a higher mortality risk.^2^ Liver cirrhosis showed the highest correlation with mortality (Table 1). Our patient had not had recent contact with the healthcare system; therefore, she likely acquired the infection in her community, and the only risk factor we could identify was her history of liver cirrhosis.

**TABLE 1:** Univariate and multivariate analyses of risk factors associated with mortality in patients with *S. maltophilia* bacteremia.

	OR	95% CI
Cancer	1.422	0.34–5.98
Liver cirrhosis	7.833	1.28–47.96
Liver metastases	4.5	0.851–23.8
polymicrobial	6.125	0.35–108.1

*Note*: Reprinted from Chang et al. *Stenotrophomonas maltophilia* bloodstream infection: comparison between community‐onset and hospital‐acquired infections. Journal of Microbiology, Immunology and Infection. 2014 February; 47(1):28–35.

Abbreviations: CI, confidence interval; OR, odds ratio.

The susceptibility of patients with liver cirrhosis to infections with *S. maltophilia* is not entirely understood. Liu et al. observed intrahepatic reduced microbial diversity in hepatocellular carcinoma (HCC). Furthermore, the HCC microbiota in patients with cirrhosis showed a higher abundance of *S. maltophilia*. In mice models, *S. maltophilia* provoked senescence‐associated secretory phenotype (SASP) in hepatic stellate cells (HSCs) by activating the TLR‐4‐mediated NF‐κB signaling pathway, which induced NLRP3 inflammasome complex formation with the secretion of various inflammatory factors. Moreover, signs of SASP were also observed in the HSCs in the HCC area with higher *S. maltophilia* enrichment in patients with cirrhosis.^4^ These results may help describe the relationship between *S. maltophilia* infection and liver diseases.

Treatment of *S. maltophilia* infection can be complicated because the bacterium has several molecular mechanisms that facilitate the acquisition of resistance to multiple broad‐spectrum antibiotics.^5^ According to the 2022 Infectious Diseases Society of America (IDSA) guidelines, a “standard of care” for *S. maltophilia* infections is not yet available. For mild cases, one can consider using trimethoprim‐sulfamethoxazole (TMP‐SMX), minocycline (MINO), tigecycline, and levofloxacin (LVFX), or cefiderocol, all as monotherapy. TMP‐SMX is still regarded as the first‐line treatment. In moderate to severe disease, any of three approaches are suggested^1^: the use of combination therapy, with TMP‐SMX and minocycline as the favored combination^2^; the initiation of TMP‐SMX monotherapy with the addition of a second agent (minocycline [preferred], tigecycline, and levofloxacin, or cefiderocol) if there is a delay in clinical improvement with TMP‐SMX alone; or^3^ the combination of ceftazidime‐avibactam and aztreonam, when intolerance or inactivity of other agents are anticipated.^6^ Of note, the IDSA panel recommends to use of LVFX “with caution” because *S. maltophilia* resistance mechanisms (e.g., efflux pumps, Sm*qnr* genes) that could inactivate the antibiotic.

**FIGURE 3:**
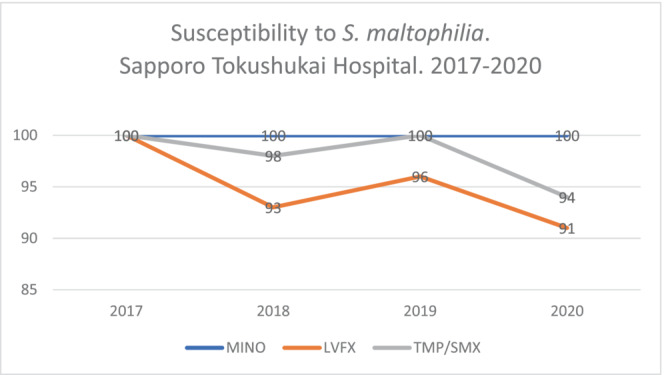
Susceptibility to *S. maltophilia*. Sapporo Tokushukai Hospital (2017–2020).

A 2014 study of *S. maltophilia* in vitro antibiotic susceptibility found that the bacteria were more susceptible to LVFX or MINO than to TMP‐SMX.^2^ In our institution, a 4‐year (2017–2020) review of *S. maltophilia* susceptibility revealed a declining trend for LVFX and TMP‐SMX, whereas MINO still had the highest susceptibility at 100% (Figure 3). Our findings are consistent with the findings of Chang et al.

Our patient had liver cirrhosis which might be a predisposing factor for *S. maltophilia* infection. She did not have recent contact with the healthcare system. Since this bacterium is present in many environments, it was difficult to determine the exact location of her exposure. Regarding her antibiotic choice, in Japan, intravenous TMP‐SMX is not easily available as the national insurance does not cover it. Therefore, the patient received LVFX monotherapy. In retrospect, given the severity of her presentation, combination therapy with MINO was likely indicated.

## 4 CONCLUSIONS

In conclusion, *S. maltophilia* not only causes hospital‐acquired infections but is also emerging as a pathogen in community settings. Although community‐onset infection is still rare and might have lower mortality, this antibiotic‐resistant bacterial species should be considered a possible pathogen in patients having predisposing factors such as pulmonary diseases, hematologic malignancies, and probably also in patients with liver cirrhosis. Furthermore, based on the study by Chang et al., and in our institutional data, MINO might become a first‐line treatment in the future.

## AUTHOR CONTRIBUTIONS

**Kentaro Shirakura:** Writing – original draft; writing – review and editing. **Masaji Saijo:** Supervision. **Tatsuya Yamada:** Supervision. **Myeongcheol Song:** Supervision. **Shadia Constantine:** Supervision.

## FUNDING INFORMATION

The author(s) received no financial support for the research, authorship, and/or publication of this article.

## CONFLICT OF INTEREST STATEMENT

The authors declare that they have no competing interests.

## CONSENT

Written informed consent was obtained from the patient to publish this report in accordance with the journal's patient consent policy.

## Data Availability

Not applicable.

## References

[1] Flores‐Treviño S , Bocanegra‐Ibarias P , Camacho‐Ortiz A , Morfín‐Otero R , Salazar‐Sesatty HA , Garza‐González E . *Stenotrophomonas maltophilia* biofilm: its role in infectious diseases. Expert Rev Anti Infect Ther. 2019;17:877‐893. 31658838 10.1080/14787210.2019.1685875

[2] Chang YT , Lin CY , Lu PL , et al. *Stenotrophomonas maltophilia* bloodstream infection: comparison between community‐onset and hospital‐acquired infections. J Microbiol Immunol Infect. 2014;47(1):28‐35. 23040236 10.1016/j.jmii.2012.08.014

[3] Falagas ME , Kastoris AC , Vouloumanou EK , Dimopoulos G . Community‐acquired *Stenotrophomonas maltophilia* infections: a systematic review. Eur J Clin Microbiol Infect Dis. 2009;28:719‐730. 19224257 10.1007/s10096-009-0709-5

[4] Liu B , Zhou Z , Jin Y , et al. Hepatic stellate cell activation and senescence induced by intrahepatic microbiota disturbances drive progression of liver cirrhosis toward hepatocellular carcinoma. J Immunother Cancer. 2022;10(1):e003069. 34996812 10.1136/jitc-2021-003069 PMC8744134

[5] Tanimoto K . *Stenotrophomonas maltophilia* strains isolated from a university hospital in Japan: genomic variability and antibiotic resistance. J Med Microbiol. 2013;62(PART4):565‐570. 23264453 10.1099/jmm.0.051151-0

[6] Tamma PD , Aitken SL , Bonomo RA , Mathers AJ , van Duin D , Clancy CJ . Infectious Diseases Society of America guidance on the treatment of AmpC β‐lactamase–producing Enterobacterales, carbapenem‐resistant *Acinetobacter baumannii*, and *Stenotrophomonas maltophilia* infections. Clin Infect Dis. 2021;74:2089‐2114. 10.1093/cid/ciab1013 34864936

